# Quality and Microbial Changes in Omega-3-Enriched Rabbit Meat Packaged with an Active Absorbent Pad in MAP

**DOI:** 10.3390/foods14030404

**Published:** 2025-01-26

**Authors:** Marta Castrica, Michela Contò, Nour Elhouda Fehri, Giulio Curone, Claudia M. Balzaretti, Egon Andoni, Alda Quattrone, Daniele Vigo, Stella Agradi, Laura Menchetti, Olimpia Barbato, Dino Miraglia, Gabriele Brecchia, Sebastiana Failla

**Affiliations:** 1 Department of Comparative Biomedicine and Food Science, University of Padova, Agripolis, Viale dell’Università 16, 35020 Legnaro, Italy; marta.castrica@unipd.it; 2 Consiglio per la Ricerca in Agricoltura e l’Analisi Dell’Economia Agraria (CREA), Centro di Ricerca Zootecnia e Acquacoltura, Research Centre for Animal Production and Aquaculture, Via Salaria 31, 00015 Rome, Italy; michela.conto@crea.gov.it (M.C.); sebastiana.failla@crea.gov.it (S.F.); 3 Department of Veterinary Medicine and Animal Sciences, University of Milan, Via dell’Università 6, 26900 Lodi, Italy; giulio.curone@unimi.it (G.C.); claudia.balzaretti@unimi.it (C.M.B.); alda.quattrone@unimi.it (A.Q.); daniele.vigo@unimi.it (D.V.); gabriele.brecchia@unimi.it (G.B.); 4 Faculty of Veterinary Medicine, Agricultural University of Tirana, Kodër Kamëz, 1029 Tirana, Albania; eandoni@ubt.edu.al; 5 Department of Veterinary Sciences, University of Torino, Largo Paolo Braccini 2, 10095 Grugliasco, Italy; stella.agradi@unito.it; 6 School of Biosciences and Veterinary Medicine, University of Camerino, Via Circonvallazione 93/95, 62024 Matelica, Italy; laura.menchetti@unicam.it; 7 Department of Veterinary Medicine, University of Perugia, Via San Costanzo 4, 06126 Perugia, Italy; olimpia.barbato@unipg.it (O.B.); dino.miraglia@unipg.it (D.M.)

**Keywords:** rabbit meat, active pad, microbial, lipid oxidation, linseed, *Padina pavonica*

## Abstract

This study evaluated the efficacy of an active absorbent pad (*a*PAD) in reducing microbial growth and enhancing the shelf life of rabbit meat stored in modified atmosphere packaging (MAP). Thigh muscles from 60 rabbits were used, divided into three dietary groups: a control group (CNT), a group supplemented with 5% extruded flaxseed (ELS5%), and a group with 3.5% extruded flaxseed and 0.2% *Padina pavonica* algae (LPP3.5%). Samples were packaged in MAP (70% O_2_, 30% CO_2_) with either a conventional pad (*n*PAD) or *a*PAD and analyzed at 1, 4, 7, 14, 21 days. Microbiological analysis revealed a significantly lower total viable count at 21 days in the ELS5%*a*PAD group. For coagulase-positive staphylococci, the CNT*a*PAD group showed lower microbial counts at both day 4 and day 21 (*p* < 0.05). *Enterobacteriaceae* reductions were observed at 24 h post packaging in both the CNT*a*PAD and LPP3.5%*a*PAD groups and at day 14 in ELS5%*a*PAD. Lipid oxidation (TBARS) was also lower in *a*PAD samples, particularly in LPP3.5%, which remained below 1.5 mg MDA/kg compared to >2.5 mg MDA/kg in *n*PAD (*p* < 0.05). Sensory attributes such as texture and color were better preserved with *a*PAD. These findings underscore the effectiveness of *a*PAD in MAP to control microbial growth, limit oxidation, and extend the shelf life of omega-3-enriched rabbit meat, providing a promising solution for functional meat product preservation.

## 1. Introduction

Rabbit meat production and consumption are significant in certain Mediterranean countries, where cultural and traditional factors strongly influence its demand [1,2,3]. Renowned for its nutritional profile, rabbit meat serves as a high-quality source of protein, characterized by low levels of cholesterol and saturated fats. Furthermore, it provides a rich source of polyunsaturated fatty acids, B vitamins, as well as minerals [4,5]. Its high digestibility and low caloric content make it especially appealing to health-conscious consumers seeking functional foods [4,6,7]. However, despite these nutritional advantages, cultural perceptions often limit its broader acceptance and consumption.

Rabbit meat also exhibits significant potential as a functional food through dietary enrichment with bioactive compounds [8,9,10,11]. Recent studies have explored the inclusion of nutraceuticals, such as flaxseed and algae-derived products, in rabbit diets. These enrichments have demonstrated substantial improvements in meat quality, particularly by enhancing the n-3 PUFA content [12,13,14,15,16]. The use of macroalgae in animal feed has also been shown to support gut health due to the presence of bioactive compounds with prebiotic and immunomodulatory properties [17,18,19]. The antioxidative capacity of macroalgae, attributed to their abundance of polyphenols and α-tocopherols, has shown promise in mitigating inflammatory and microbial challenges while preserving the sensory and nutritional quality of meat [20,21,22]. The lack of published data on its application in rabbit diets or its effects on meat quality and shelf life presents an opportunity for further investigation.

The shelf life of rabbit meat is critically influenced by biochemical and microbiological processes, notably lipid oxidation and microbial proliferation. During storage, fresh meat releases exudates, creating a favorable environment for microbial growth due to its high water activity and rich nutrient composition [23]. This environment significantly reduces the meat’s shelf life and commercial value, contributing to substantial economic losses during the production, retail, and storage stages. Addressing the rapid oxidative degradation of PUFAs is essential, as it not only leads to rancidity but also adversely impacts the sensory and nutritional qualities of the meat [24,25,26]. Consequently, interventions aimed at prolonging shelf life are imperative for ensuring product quality and minimizing economic losses.

Modified atmosphere packaging (MAP) has emerged as a key technology to enhance the preservation of fresh meat. By replacing ambient air with tailored gas mixtures, MAP effectively slows microbial growth and oxidative processes [27,28]. In red meats, oxygen concentrations are crucial for maintaining a desirable appearance; however, for rabbit meat, which is a white meat, the esthetic requirement for oxygen is less critical [28]. Nonetheless, research indicates that oxygen levels of 60% or higher in MAP mixtures can yield favorable preservation outcomes for rabbit meat. In this context, Racewicz et al. [29] demonstrated that an oxygen concentration of 70% significantly reduced the *Enterobacteriaceae* population in rabbit meat samples after 21 days of storage. Moreover, several authors showed that the incorporation of carbon dioxide in the range of 20–40% further inhibits aerobic bacteria by extending microbial lag phases and increasing doubling times [29,30,31]. Such gas mixtures not only maintain the fresh appearance of meat but also significantly delay spoilage.

Innovative packaging technologies, such as active packaging systems, have further advanced the preservation of perishable foods [1,27,32]. These systems, defined by EU Regulation No. 450/2009, involve the integration of components that interact with the packaged food or its environment to extend shelf life and preserve quality.

Recent advancements focus on developing and enhancing absorbent pads with active and bio-based components to provide antimicrobial and antioxidant properties. Several researchers have conducted studies on this topic in recent years; Sun et al. [33] developed potassium-doped sodium alginate hydrogel pads that effectively absorbed exudates and inhibited spoilage in chilled pork, extending its shelf life by two days. Wang et al. [34] demonstrated that absorbent pads with levulinic acid and sodium dodecyl sulfate significantly reduced microbial loads and enhanced microbial diversity in ground beef. Jiang et al. [35] introduced bio-based pads from delignified wood fibers and polyvinyl alcohol that extended pork shelf life to over nine days while reducing environmental impact. Lastly, Liu et al. [36] highlighted the efficacy of pads infused with *Carum copticum* essential oil in extending the shelf life of chicken meat through antimicrobial and antioxidant activity. When combined with MAP, active absorbent pads can play a more significant role by not only absorbing liquids but also releasing antioxidant and antimicrobial agents [37]. This dual functionality enhances meat preservation while ensuring food safety, quality, and sustainability. Despite these advancements, research on packaging solutions specifically tailored for rabbit meat remains limited, likely due to its niche market and comparatively higher production costs.

Excessive drip loss is a critical parameter affecting the commercial value of rabbit meat. Beyond reducing yield, excessive exudation adversely impacts the texture, visual appeal, and overall shelf life of the product [23]. Active absorbent pads, by effectively managing moisture levels, help maintain product freshness and minimize microbial colonization [37]. Deteriorative microbial activity accelerates protein degradation and lipid rancidity, leading to a loss of nutritional properties and adverse impacts on organoleptic attributes such as discoloration, textural changes, off-flavors, and unpleasant odors [23].

The spoilage of rabbit meat is predominantly caused by psychrotrophic Gram-negative bacteria, such as *Pseudomonas* spp. [24], which thrive in aerobic conditions. These microorganisms contribute to protein degradation, lipid hydrolysis, and the production of volatile compounds, resulting in reduced consumer acceptability. Furthermore, lipid and protein oxidation are exacerbated by the reactive oxygen species (ROS) generated during storage. Oxidative protein modifications can alter primary structures and side chains, resulting in aggregation and gelation, thereby reducing the bioavailability of essential amino acids and diminishing the meat’s nutritional value [38]. The dual role of oxygen supporting aerobic microbial growth while facilitating oxidative processes necessitates precise control over its levels within packaging systems to balance microbial inhibition with sensory preservation.

This study hypothesizes that the integration of an active absorbent pad within a MAP system could effectively reduce microbial growth and oxidative degradation, particularly in rabbit meat enriched with n-3 PUFAs through dietary supplementation with extruded flaxseed and *Padina pavonica* extract. While these supplementations in rabbit diet enhancements significantly improve the meat’s nutritional profile, they may also increase susceptibility to oxidative damage, potentially limiting shelf life. Thus, this research aims to evaluate the efficacy of an active absorbent pad in mitigating microbial proliferation and prolonging the shelf life of PUFA-enriched rabbit meat stored under modified atmosphere conditions. By addressing these challenges, the study seeks to provide innovative strategies for preserving the quality and commercial viability of rabbit meat as well as exploring its potential as a functional food.

## 2. Materials and Methods

### 2.1. Meat Samples Preparation

The experimental trial took place at Azienda Agricola Brachino Patrizia, a commercial rabbit farm situated in Central Italy. This study was conducted as part of the PRIMA project “Omega Rabbit: food for health BenefIT”, supported by funding from the European Union. The handling of animals during the trial adhered to Legislative Decree No. 146, which enforces Directive 98/58/EC.

After weaning at 35 days old, the rabbits were housed individually in standard cages (L × W × H: 75 × 38 × 25 cm) under controlled environmental conditions. They were randomly assigned to one of three experimental groups which received a specific pellet diet. The isoenergetic and isoprotein diets were briefly formulated as follows: CNT = a control diet; ELS5% = CNT diet with 5% extruded flaxseed; and LPP3.5% = CNT diet containing 3.5% extruded flaxseed and 0.2% *Padina pavonica* algae extract, as detailed in Fehri et al. [39] Fresh water was available at all times. Rabbits were slaughtered at 85 days of age, and 20 carcasses chosen randomly for each diet were used.

Both sides’ hind legs (HLs) were aseptically excised from the carcasses of rabbits in each experimental group and transported under cold chain conditions to the laboratory of CREA. Upon arrival, the two HLs for each animal were deboned and sectioned into slices, which were subsequently packaged in a modified atmosphere containing 70% O_2_ and 30% CO_2_. This high-oxygen composition is commonly used for MAP preservation of rabbit meat [29]. The packaging consisted of polystyrene trays, with the base fitted with either a control pad (*n*PAD), made of non-woven fabric designed only to absorb liquids, or an active pad (*a*PAD). This configuration was applied across all three diet groups: CNT, ELS5%, and LPP3.5%.

### 2.2. Active Absorbent Pad

The *a*PAD (trade name: “Bacteria Catcher”) employed in this study was designed and supplied by ANT Advanced Nonwovens Technologies s.r.l., a company that is part of the Deatex group based in Milan, Italy. The *a*PAD used in this trial is identical to the one reported by Castrica et al. [37]. Briefly, the *a*PAD, measuring 7.5 × 13.5 cm, comprises two main components: an absorbent section, constructed from non-woven fabric and anchored to the base of the packaging, and an active section impregnated with an additive mixture. This mixture consists of 30–50% polymeric cationic agents by weight, 10–20% base by weight, and 1–10% auxiliary substances by weight.

The active section of the *a*PAD is designed for direct contact with rabbit meat and exerts an attraction effect on bacterial cell walls. The *a*PAD analyzed in this investigation complies with Regulation (EC) No. 1935/2004 concerning materials and articles intended for food contact. Additionally, it does not fall under the classification of a biocidal product as defined by Regulation (EC) No. 528/2012, as its mechanism of action is strictly physical and mechanical.

### 2.3. Experimental Design

Then, HLs for each diet group were packaged with the *a*PAD or with the *n*PAD. Five slices obtained for each animal were packaged in the same way, and one slice was used for each experimental time.

All the packaged slices were stored at 4 ± 1 °C and analyzed at specific time points: 24 h post packaging (D1), after 4 (D4), 7 (D7), 14 (D14), and 21 days (D21) for a total of 300 samples.

**Figure 1:**
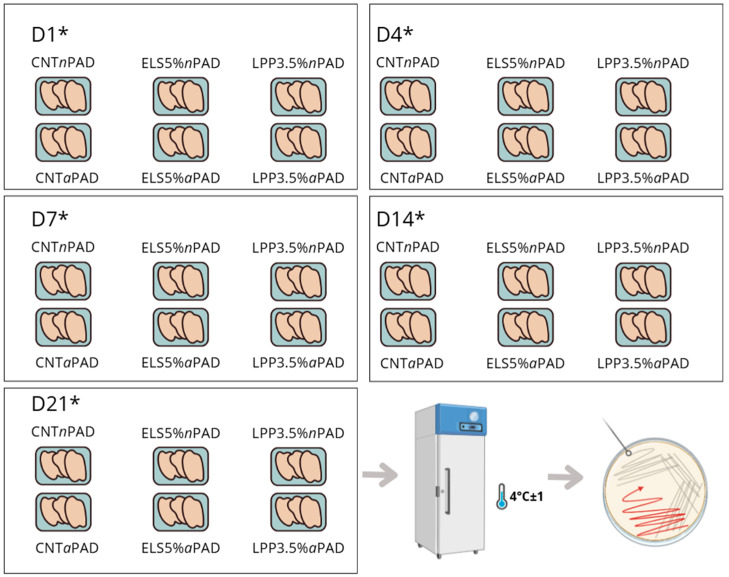
Microbiological and chemical shelf life experimental protocol. * For each analysis time point, 10 samples per group were analyzed.

Packages containing HL slices were assigned for analyses, as detailed in the experimental design, shown in Figure 1, dividing each sample into two parts: one for microbiological shelf life and the other for chemical and sensory determinations.

### 2.4. Water-Holding Capacity (WHC) and pH

The slices were weighed before packaging and then reweighed at the established time to assess the water-holding capacity (WHC), determined by the loss of liquids during storage via the following equation: (W0 − WT_1,4,7,14,21_)/W0 × 100, where W0 = weight pre-packaging and WT = weight at different storage times.

**Figure 2:**
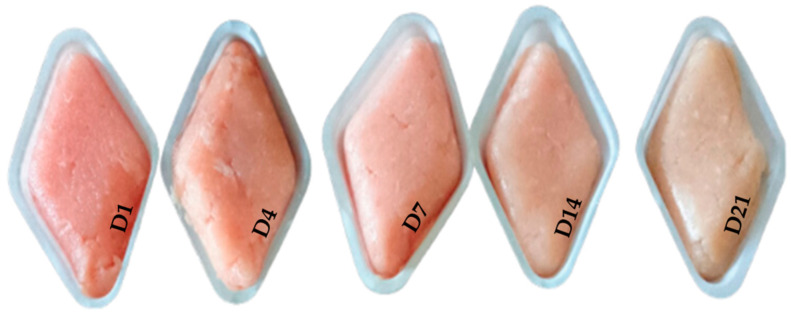
Minced fore leg meat in different storage times for sensorial test.

At each time point, the portion of the sample designated for chemical and sensory analysis was ground in an ice bath to ensure sample uniformity (Figure 2).

pH was measured using a pH meter with temperature compensation (XS Instrument Serie80 PC80, Giorgio Bormac s.r.l., Carpi, MO, Italy). One gram of meat was homogenized with 10 mL of NaCl 0.9%, and for each sample, three measurements were performed, and the final value was obtained as the mean.

### 2.5. Color

Color analyses were performed by maintaining minced meat in air for 30 min to allow for the blooming effect, and color parameters were recorded using the CIELAB system [40] to evaluate lightness (L*), redness (a*), and yellowness (b*), with D illuminant (6504 °K, daylight) using a Konica Minolta CM-3600 D (Sensing, Inc., Osaka, Japan) spectrophotometer. Chrome (C) and hue (H) were calculated using a* and b* indexes with the following equations:

(1) $$C=\sqrt{a^{2}}+b^{2};\;H={\mathrm{tang}}^{-1}b*/a*$$

### 2.6. Microbiological Analysis

Total viable counts (TVCs) for *Enterobacteriaceae*, *Escherichia coli*, and coagulase-positive staphylococci were quantified using Petrifilm (3M, St. Paul, MN, USA). *Pseudomonas* spp. were cultured on Pseudomonas Agar Base (Biolife Italiana s.r.l., Milan, Italy) with CFC Pseudomonas Supplement (Biolife Italiana s.r.l., Milan, Italy) and incubated at 25 °C for 48 h. *Brocothrix thermosphacta* was grown on STAA Agar Base (Biolife Italiana s.r.l., Milan, Italy) supplemented with STAA Selective Supplement (Biolife Italiana s.r.l., Milan, Italy) and incubated at 22 °C for 48 h.

All analyses were performed in duplicate, and results were expressed as Log CFU/g. Additionally, the detection of *Salmonella* spp. and *Listeria monocytogenes* (analytical unit: 25 g) was carried out only at D1, in accordance with UNI EN ISO 6579-1:2017 [41] and AFNOR [42] BRD 07/05-09/01 standards, with results reported as either presence or absence in 25 g of the sample.

### 2.7. Lipid Oxidation and Carbonyl and Sulfhydryl Analysis

#### 2.7.1. Thiobarbituric Acid Reactive Substance Assay (TBARS)

Lipid oxidation was quantified using the TBARS assay, with malondialdehyde (MDA) as the reference compound. Briefly, 2.5 g of meat was homogenized with water and 2.8% of ethanolic butylated hydroxytoluene (BHT) as an antioxidant. Subsequently, 1 mL of the homogenate was mixed with 1 mL of trichloroacetic acid (TCA) and centrifuged. The resulting supernatant was incubated at 80 °C for 30 min with 0.28% thiobarbituric acid (TBA) to form the MDA-TBA adduct. After cooling on ice, 10 μL of the solution was injected into an HPLC system (Alliance 2695, Waters Corporation, Framingham, MA, USA) equipped with a C18 reverse-phase column (Kinetex 5 µm EVO, Phenomenex, Torrance, CA, USA). Detection was carried out via fluorescence at λex = 515 nm and λem = 543 nm. The MDA-TBA sample peak was identified by comparison with an MDA standard peak. The TBARS concentration was expressed as mg of MDA/kg of meat, following the method described by Cifuni et al. [43].

#### 2.7.2. Sulfhydryl and Carbonyl Content

Protein oxidation was investigated in terms of changes in the sulfhydryl group and carbonyl (the CO group) content in meat. The procedures were performed as reported by Valerio et al. [44].

For the SH group analysis, 1 g of meat was homogenized with 16 mL of 20 mM potassium phosphate (pH 6.0), filtered through a gauze to remove collagen, and centrifuged. The pellet was washed twice with 16 mL of 50 mM potassium chloride, and after a 1:50 dilution, with 20 mM potassium phosphate (pH 6.0), two aliquots were used. One aliquot was mixed with 8M urea in 100 mM buffer phosphate and 10 mM 2,2-ditio bis 5-nitropiridina (DTNP), and the other was mixed only with 8M urea in 100 mM buffer phosphate, which represented the blank. Samples were incubated for 1 h in the dark, and the absorbance was measured at 386 nm with a PerkinElmer spectrophotometer Lambda 25 (PerkinElmer, Shelton, CT, USA). The sulfhydryls were expressed in nmol of SH/mg of protein.

The carbonyls were quantified from the filtrate sample obtained from 1 g of meat for sulfhydryl analysis before the dilution phase. Two aliquots of 1 mL were used. After centrifugation, one pellet was used as the blank, mixed with 1 mL of 2 N HCl, and the other pellet was treated with 1 mL of 0.2% dinitro-phenylhydrazine (DNPH) in HCl 2 N (*w*/*v*). Samples were incubated for 1 h at room temperature, and after centrifugation, the pellets were collected. The pellets were washed and centrifuged three times with ethanol:ethyl acetate (1:1 *v*/*v*) to remove the DTNP traces and solubilized lipid. After the washes, the pellets were solubilized in 6M guanidine HCl and then incubated for 1 h at 90 °C. The blank samples were used to calculate the protein concentration in the sample by measuring the absorbance at 280 nm with the PerkinElmer spectrophotometer Lambda 25 (PerkinElmer, Shelton, CT, USA), using BSA as the standard. The samples treated with DNPH were read at 370 nm. The carbonyls were expressed in nmol of DNPH/mg of protein.

### 2.8. Sensory Tests

A sensory test was conducted by 10 semi-trained panelists. In each panel session, panelists evaluated the color, odor, and overall acceptability using a hedonic scale from 0 to 10 (0 = dislike for color/odor/overall acceptability; 10 = excellent for color/odor/overall judgment) for each experimental diet (CNT, ELS 5%, and LPP 3.5%), PAD type, and storage time (D1–D21). The samples were presented as minced raw meat in white sample holders (Figure 2), and they were evaluated in an isolated room under artificial lighting. During each panel session, one sample from each storage time belonging to the same diet but with two different PADs was presented simultaneously. Each day, three panel sessions were conducted, one for each experimental diet. The sensory test was carried out over ten consecutive days, with three sessions held each day.

### 2.9. Statistical Analysis

The statistical analysis in this study was performed using the PROC MIXED procedure in SAS/STAT Software Version 9.4 (SAS Institute Inc., Cary, NC, USA). The model included the feed group, PAD type, and storage time as fixed effects, while the animal was included as a random effect. For the sensory analysis, the panelist and session analysis were considered as a random effect to account for variability among individual assessors. All possible two-way and three-way interactions between the fixed effects were tested and included in the model if they were statistically significant. Post hoc comparisons of the means were carried out using Tukey’s test, with a significance level set at *p* < 0.05. To ensure the reliability and validity of the sensory test, a comprehensive analysis of the distribution of sensory scores was conducted. Both distribution analysis and box plot analysis were employed for each sensory attribute to detect potential outliers and assess the homogeneity of scores among panelists. Descriptive statistics, including mean, median, standard deviation, and range, were calculated to evaluate the central tendency and the dispersion of sensory scores. In addition, histograms were generated for each sensory attribute to visualize the distribution and assess the normality of the data. The results of these analyses are reported in Figures S1 and S2 and Tables S1 and S2, which provide a graphical representation of the score distributions for each sensory parameter.

## 3. Results and Discussion

### 3.1. *pH and WHC*

The pH and WHC of rabbit meat during storage are critical indicators of its quality and shelf life. Their interaction provides valuable insights into the physiological and biochemical changes occurring in the meat and helps us understand the impact of storage conditions and dietary supplementation [26].

The average pH across the three feed groups was 5.97 ± 0.02 as mean ± standard error (SE), with no significant differences detected among them. Relatively high pH values around 6.0 did not appear to pose issues for the shelf life of rabbit meat, as reported by Pereira et al. [24]. However, when considering the overall effect of the PADs on pH, the *n*PAD group demonstrated a significantly lower value compared to the *a*PAD group. Notably, this difference was specifically observed in the CNT group, as highlighted in Table 1, which presents the interaction effects among the feed groups (CNT, ELS 5%, and LPP 3.5%) and the two PAD conditions on physical parameters. This difference suggests that *a*PAD effectively minimizes pH decline, potentially reducing microbial activity responsible for producing acidic metabolites [37]. For pH, the CNT*n*PAD group exhibited a significantly lower value compared to the CNT*a*PAD group (5.95 ± 0.02 vs. 6.00 ± 0.02, respectively; *p* = 0.028).

Regarding WHC, as observed for pH, the PAD effect was evident (*p* < 0.001); the *a*PAD group generally exhibited lower values compared to the *n*PAD group (2.81 ± 0.03% vs. 2.96 ± 0.03%). The water loss was positively influenced by the use of the *a*PAD, likely due to its ability to minimize liquid loss [37]. Among feed groups, ELS5% showed significantly lower values compared to the other two (*p* < 0.001). With respect to the interaction between feed and pad types, significant differences were observed exclusively within the LPP3.5% group, where the *n*PAD group demonstrated higher water loss compared to the *a*PAD group (3.08 ± 0.05% vs. 2.84 ± 0.05%; *p* < 0.001).

pH and WHC were significantly influenced by the storage time (Table 2). The pH values exhibited a significant increase at the end of the storage period, rising from 5.96 on D1 to 6.05 on D21 (*p* = 0.002). This significant increase at 21 days is consistent with proteolytic activity typically exacerbated during extended storage periods, resulting in the release of alkaline compounds such as ammonia and amines [38]. The WHC constantly increased at each time point (*p* < 0.001), particularly from D1 to D4 (+1.33%). Protein denaturation over time during aging is closely linked to an increase in liquid loss [25,45,46], further emphasizing the importance of managing these parameters to preserve the quality and shelf life of rabbit meat.

### 3.2. Color

Meat color is one of the primary sensory parameters influencing consumer choice [1,2]. Primarily governed by myoglobin, meat color is subject to changes driven by the auto-oxidation of this heme protein as well as interactions with lipid and protein oxidation processes [29,38,46].

The color, in general, was not influenced by dietary group, showing no significant differences for most parameters, except for L* (*p* = 0.038) and a* (*p* = 0.041). Specifically, the CNT group, compared to the LPP3.5% group, exhibited lower L* (60.77 ± 0.33 vs. 61.94 ± 0.33) and higher a* (1.69 ± 0.14 vs. 1.09 ± 0.14, respectively), while the ELS5% group displayed intermediate values. Regarding the color coordinates, no significant differences were observed overall in relation to the presence or absence of an active PAD. Notably, the effect of *a*PAD was not evident within the different dietary groups. During storage, fluctuations were observed in most color parameters, except for a* and H, which remained significantly unaffected by storage times. The other color parameters (L*, b*, and C) increased up to D14, likely due to enzymatic proteolytic processes that temporarily enhance the meat’s appearance by improving light reflection [46,47]. The subsequent significant decline on D21 (*p* < 0.001), indicating the onset of degradative oxidative processes. The gradual loss of color, transitioning to gray-brown, is associated with the degradation of structural and cytoplasmic proteins, including myoglobin, which is responsible for the characteristic color of fresh meat [47,48,49]. In rabbit meat, lipid and protein oxidation has been reported to contribute to discoloration, demonstrating a close relationship between oxidative processes and chromatic changes [9,29,50].

The trends observed in pH, WHC, and color highlight their interdependence. Higher pH and WHC values, as observed with the active absorbent pad, are associated with improved color, likely due to reduced oxidative and proteolytic degradation [37,38,46,51]. This oxidative protection is also evident, albeit to a lesser extent, in animals fed a diet supplemented with *Padina pavonica*. The presence of polyphenols and active compounds in this diet likely acted as scavengers, enhancing the oxidative state of the animal and, consequently, the meat quality [19,22].

### 3.3. Microbiological Profile

**Figure 3:**
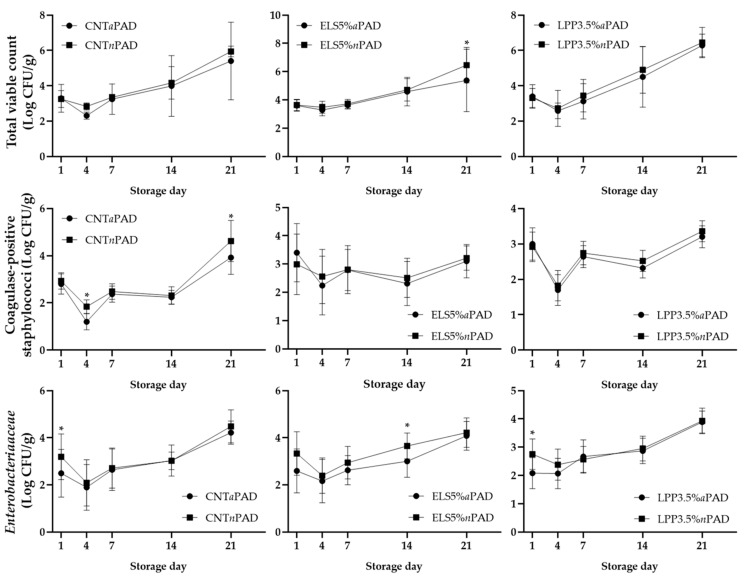
Trend of microbial development across storage days. The bar graphs show mean ± standard deviation. *: *p* < 0.05.

After 21 days of shelf life, the storage time had a significant effect on all evaluated microorganisms, with an overall increase in bacterial counts observed over time (*p* < 0.005). Moreover, significant differences at 21 days of storage were observed for coagulase-positive staphylococci, where the control group with the active PAD exhibited lower microbial loads compared to the CNT group with the *n*PAD (*p* < 0.001). Notably, the slowdown, likely due to the effect of the *a*PAD, was already evident by day 4 (Figure 3). A similar trend was observed for the total viable count, where the ELS5% *a*PAD group exhibited lower counts compared to the ELS5%*n*PAD group at day 21. This finding aligns with the observations reported by Cullere et al. [1], where the group fed a diet supplemented with flax exhibited a reduction in the total bacterial count.

**Figure 4:**
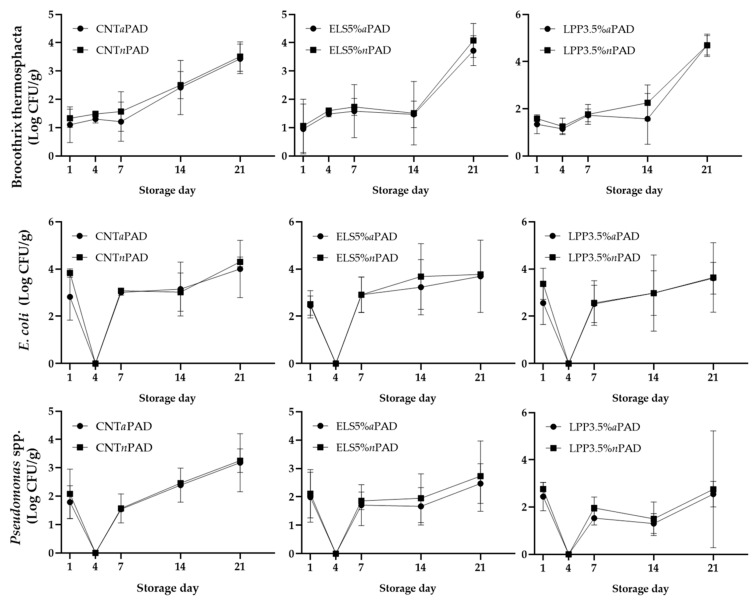
Trend of microbial development across storage days. The bar graphs show mean ± standard deviation. *p* < 0.05.

For *Enterobacteriaceae*, lower microbial loads were already evident at D14 in the ELS5% *a*PAD group compared to the ELS5% *n*PAD group (*p* < 0.001). Furthermore, for this microorganism, differences became apparent as early as 24 h of storage in both the CNT and LPP3.5% groups, with packages containing *n*PAD exhibiting higher values compared to those with *a*PAD. These differences, observed even in the early days of shelf life, could be attributed to the hygienic conditions in the slaughterhouse and during processing [52]. The variations identified from day 14 onward can be linked to the selective effects of different factors, including temperature, pH, and packaging atmosphere, on the predominant bacterial populations and spoilage dynamics during storage [1,53]. In the present study, the average pH values recorded across all experimental groups remained at or below 6. Such low pH levels are recognized for their bacteriostatic properties, which help maintain microbial equilibrium and contribute to the preservation of meat quality [54]. Finally, no significant effect was observed against *Brochothrix thermosphacta*, *Pseudomonas* spp., and *E. coli* throughout the storage days (Figure 4). The findings of this study regarding the antimicrobial pad align with those of Komodromos et al. [55] and Fernandez et al. [56] who observed no statistically significant impact of the antimicrobial pad on the counts of *Pseudomonas* spp. and *B. thermosphacta*. A similar conclusion can also be drawn for *E. coli* in the present investigation. Moreover, when evaluating the results on the antimicrobial efficacy of the pad, it is essential to consider the substantial differences between the pads tested in this study and those reported in the literature [33,34,35,36,55,56]. These differences, primarily influenced by variations in composition, may result in significantly distinct actions depending on the bacterial species targeted or the specific parameter under investigation.

All samples were negative for *Salmonella* spp. and *L. monocytogenes*. The present study investigated the interplay between diet and packaging method, with particular attention to the effectiveness of MAP in comparison to air-permeable overwrapping for preserving meat quality over longer storage durations. According to Bobbitt [57], the shelf life of rabbit carcasses stored in aerobic conditions ranges between 3 and 6 days, whereas MAP storage can extend this period by up to threefold. This extension in shelf life can be attributed to the role of the gaseous environment, alongside factors such as meat pH and storage temperature, in shaping the microbial communities. For instance, it is widely recognized that carbon dioxide sensitivity in pseudomonads and *Enterobacteriaceae* [58] influences microbial growth [1]. In contrast, MAP tends to favor the proliferation of facultative anaerobes such as *Brochothrix thermosphacta* as the main spoilage microorganisms [30,59]. In the current study, sensory evaluations indicated that rabbit meat retained acceptable quality levels until day 14. This observation suggests a synergistic preservative effect arising from the combined influence of diet and packaging. Rodríguez-Calleja et al. [60] noted that rabbit meat stored under vacuum conditions reached unacceptable sensory scores by day 28. Similarly, findings by Berruga et al. [30] indicate that under various MAP gas compositions, lactic acid bacteria typically exhibit a lag phase of approximately 10 days.

### 3.4. Lipid Oxidation and Carbonyl and Sulfhydryl Grouphiol

**Figure 5:**
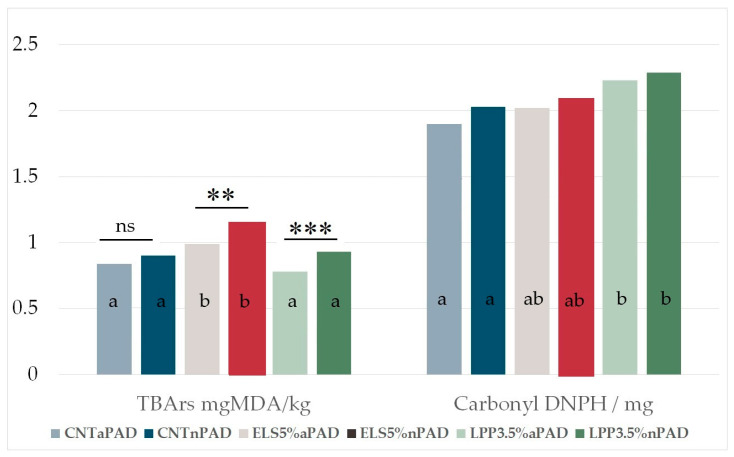
TBARS and the carbonyl group of rabbit meat affected by different diets and pads on average for different stored times. Significant differences between *a*PAD and *n*PAD inside the same feed group are indicated as ns: not significant, ** *p* < 0.01, and *** 0.001; different letters mean significant differences among (*p* < 0.05) the means of the diets.

TBARS levels are widely used to assess lipid peroxidation, reflecting the formation of secondary oxidative products like malondialdehyde (MDA). In rabbit meat, the enrichment of PUFA n-3, as achieved through dietary supplementation with linseed or algae, increases its susceptibility to lipid oxidation due to the higher number of double bonds in PUFA [61,62]. In terms of oxidation parameters, the TBARS value (Figure 5) was significantly higher (*p* < 0.001) in the ELS5% group (1.07 ± 0.04 mg MDA/kg) compared to the CNT and LPP3.5% groups (0.86 ± 0.04 mg MDA/kg; on average for the two groups). The presence of active PAD significantly reduced TBARS value (*p* = 0.005). With the exception of CNT, the other two groups displayed lower TBARS levels in *a*PAD compared to *n*PAD (0.99 vs. 1.15 mg MDA/kg for ELS5% and 0.78 vs. 0.93 mg MDA/kg for LPP3.5% with error standard 0.05).

The carbonyl group (C=O) is a polar group because oxygen is more electronegative than carbon, and it is formed when reactive oxygen species (ROS) attack amino acid side chains, leading to oxidative modifications in proteins. Protein oxidation, as evidenced by elevated carbonyl levels, contributes to structural changes that affect the texture and WHC of rabbit meat. For carbonyls, the CNT group exhibited significantly lower values than the LPP3.5% group (*p* < 0.001), while no significant differences were observed for the PAD type. The polyphenols from algal supplementation were expected to counteract the formation of oxidation products [22,37]. However, the elevated levels of long-chain polyunsaturated fatty acids (PUFAs) in rabbit meat, from animals fed with extruded linseed and *Padina pavonica*, as highlighted by Agati et al. [63], exhibited a heightened susceptibility to oxidation, thereby diminishing the protective efficacy of the algal polyphenols.

The sulfhydryl group showed no significant differences attributable to either the diet or the PAD type. The sulfhydryl group (-SH) is a sensitive marker of oxidative damage in proteins, primarily cysteine residues, which are critical for maintaining protein structure and enzymatic function. The loss of the sulfhydryl group is indicative of disulfide bond formation, leading to protein cross-linking and aggregation [38].

Over time, both TBARS and carbonyl levels increased significantly at each analysis storage point. TBARS levels rose from 0.06 ± 0.31 at D1 to 2.15 ± 0.31 mg MDA/kg at D21 (Table 3), while carbonyl levels increased from 0.67 ± 0.44 at D1 to 3.62 ± 0.44 nmol of DNPH/mg of protein at D21. In contrast, the sulfhydryl group, because it acts as a scavenger for oxidative processes, exhibited a significant decrease over time, declining from 69.11 ± 5.65 nmol SH/mg protein at D1 to 32.27 ± 5.65 nmol SH/mg protein at D21 (*p* < 0.001).

The interplay between TBARS and the carbonyl and sulfhydryl groups underscores the oxidative dynamics in rabbit meat during storage. While lipid oxidation primarily affects flavor and aroma, protein oxidation alters texture, WHC, and appearance. The active pad’s role in mitigating both lipid and protein oxidation, along with dietary strategies such as supplementation with *Padina pavonica*, demonstrates an integrated approach to enhancing the oxidative stability and overall quality of rabbit meat, particularly for products enriched with PUFA n-3 [1,29].

### 3.5. Sensory Traits

The panelists’ evaluations revealed significant differences for color and overall acceptability attributes exclusively in the ELS5% group, where the ELS5%*a*PAD samples received higher scores compared to the ELS5%*n*PAD samples (color, *p* = 0.006 and overall acceptability, *p* = 0.009; Table 4).

Across all groups, irrespective of the presence or absence of an active PAD, samples received higher scores during the initial days of shelf life. However, as the shelf life progressed, the scores (Table 5) for all evaluated attributes declined significantly over time (*p* < 0.001), reflecting the cumulative impact of oxidative and microbial spoilage on sensory attributes. Sensory evaluations of color and odor further reinforced the importance of an active PAD in maintaining meat quality. The observed sensory deterioration aligns with microbial spoilage, a critical factor contributing to discoloration and the development of abnormal colors in meat [64].

### 3.6. Interaction of Principals Parameters

**Figure 6:**
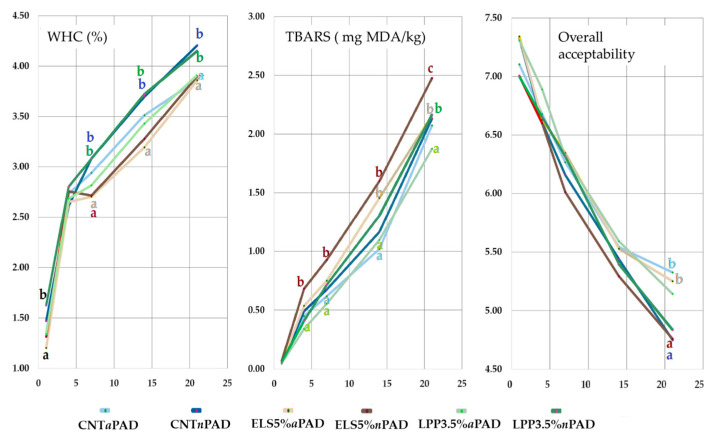
Trend of water-holding capacity (WHC). TBARS and overall acceptability across the storage times. Different letters at the same time mean significant differences for *p* < 0.05; the color of the letter corresponds to the color of the group; the group without letters is in the intermediate position.

Some of the most significant interactions between the feed groups, PAD, and storage time are illustrated in Figure 6. Considering the WHC, significant differences were observed early at D1 of storage between ELS5%*a*PAD and LPP3.5%*n*PAD (1.20 ± 0.10% vs. 1.63 ± 0.10%), with the latter showing the highest value among all groups. These differences between the two groups persisted throughout the 21 days of storage, except on D4, when all groups exhibited a sudden increase in liquid loss. Subsequently, regardless of the PAD type, the ELS5% group consistently presented the lowest liquid loss, significantly different from both LPP3.5%*n*PAD and CNT*n*PAD. The other groups exhibited intermediate values, with differences remaining constant over time, even as liquid loss increased.

Considering TBARS, all groups showed similar values at D1 (0.06 mg MDA/kg of meat). However, by D4, the ELS5% *n*PAD group was significantly different from the LPP3.5%*a*PAD group (0.68 ± 0.10 mg MDA/kg vs. 0.34 ± 0.10 mg MDA/kg). At D7, while maintaining the same differences observed at D4, the CNT*a*PAD and LPP3.5%*a*PAD groups showed the lowest TBARS values (0.58 ± 0.10 mg MDA/kg). By D14, the ELS5%*a*PAD group also exhibited high TBARS values, similar to samples with the normal PAD, significantly differing from other active PAD samples. At D21, the LPP3.5%*a*PAD group presented the lowest TBARS values compared to the others at the same time, while the ELS5%*n*PAD group showed the highest (1.87 vs. 2.48 mg MDA/kg, respectively), followed by ELS5% *a*PAD and LPP3.5%*n*PAD (2.16 mg MDA/kg on average). This suggests that the synergistic effect of algae polyphenols and the active PAD remains effective in controlling lipid oxidation over extended storage periods, highlighting its potential for enhancing the oxidative stability of PUFA-enriched rabbit meat [22]. In contrast, the ELS5%*n*PAD group showed the highest TBARS values, emphasizing the heightened susceptibility of linseed-enriched diets to lipid oxidation in the absence of adequate antioxidant interventions. The remaining groups with the active PAD showed no significant differences among themselves, displaying the lowest TBARS values overall. This indicates that the protective effect of the active PAD diminishes over prolonged storage, particularly in groups with higher PUFA content, such as those fed with extruded linseed [61].

Regarding overall acceptability, which summarizes the sensory evaluation of the color and odor of raw meat, differences among the groups were only evident at D21. Samples stored without the active PAD exhibited lower acceptability, particularly for CNT (4.75) and ELS5% (4.84). Conversely, samples stored with the active PAD received significantly higher evaluations, particularly for CNT and ELS5% (5.33 and 5.18). Nevertheless, overall sensory acceptability declined significantly over time for all six groups analyzed. In addition to oxidation reactions, microbial spoilage is another significant factor underlying the sensory deterioration of meat [64], causing discoloration and the development of abnormal colors.

## 4. Conclusions

The present study underscores the efficacy of an active absorbent pad in enhancing the shelf life of rabbit meat, particularly in the context of omega-3 polyunsaturated fatty acid (PUFA) enrichment achieved through dietary supplementation with extruded linseed and *Padina pavonica*. The inclusion of *a*PAD within modified atmosphere packaging effectively mitigated microbial growth and oxidative degradation, as evidenced by reduced TVCs, lipid peroxidation (TBARS), and protein oxidation indicators such as carbonyl and sulfhydryl content.

The oxidative stability of rabbit meat was significantly improved with the use of *a*PAD, particularly in the LPP3.5% dietary group, which exhibited the lowest TBARS values and enhanced sensory scores after extended storage. This suggests that the polyphenols and bioactive compounds in *Padina pavonica*, combined with the physical and chemical properties of *a*PAD, play a synergistic role in preserving meat quality. However, the elevated levels of long-chain PUFAs in enriched meat heightened its susceptibility to oxidative damage, underscoring the challenges associated with balancing nutritional enhancement and oxidative stability.

WHC and pH trends further emphasized the role of *a*PAD in maintaining the physicochemical properties of rabbit meat, with higher WHC and stable pH values correlating with reduced microbial and oxidative activity. These parameters also showed a clear interdependence with meat color stability, where active packaging systems contributed to the preservation of visual appeal, a metric crucial for consumer acceptance. Despite these advances, this study highlights the need for further exploration of optimal combinations of dietary strategies and packaging technologies to fully realize the benefits of omega-3 enrichment while minimizing the risks of oxidation. The findings offer valuable insights into innovative preservation methods, demonstrating the potential of *a*PAD as a practical and effective solution for improving the shelf life and quality of functional meat products.

## 5. Patents

Principi, A. and Merlotti, S. (2022). “International Patent application PCT WO 2022/029597A1—Bacteria—trapping item”.

The complete disclosure could be forwarded to readers but only under an NDA agreement.

## Figures and Tables

**Table 1:** Physical parameters of rabbit meat affected by different diets and PADs in different stored times.

	CNT		ELS5%		LPP3.5%				
	*n*PAD	*a*PAD	*n*PAD	*a*PAD	*n*PAD	*a*PAD	p Diets	p Pad	RMSE
pH	5.93 ^a^	6.07 ^b^	5.98	5.97	5.96	5.97	0.423	0.028	0.14
WHC%	3.01	2.88	2.79	2.72	3.08 ^b^	2.84 ^a^	<0.001	<0.001	0.31
L*	60.99 ^a^	60.56 ^a^	61.38 ^ab^	61.85 ^ab^	61.73 ^b^	62.16 ^b^	0.038	0.670	2.07
a*	1.68 ^b^	1.71 ^b^	1.54 ^ab^	1.58 ^ab^	1.29 ^a^	0.90 ^a^	0.041	0.495	0.54
b*	5.22	4.67	5.31	5.44	5.04	4.84	0.121	0.339	1.19
C	5.59	5.12	5.63	5.80	5.31	5.01	0.094	0.380	1.54
H	72.20	69.92	73.88	73.84	75.68	79.50	0.371	0.637	11.52

CNT = control diet; ELS5% = CNT diet with 5% extruded flaxseed; LPP3.5% = CNT diet containing 3.5% extruded flaxseed and 0.2% *Padina pavonica* algae extract; *n*PAD= control pad; *a*PAD = active pad; WHC = water-holding capacity; L* = lightness; a* = redness index; b* = yellowness index; C = chrome; H = hue; RMSE = root square error; ^a,b^ = different letters in the same row indicate a significant difference for *p* < 0.05; data are expressed as the mean.

**Table 2:** Effect of different storage times and PADs on the physical characteristics of rabbit meat.

	D1	D4	D7	D14	D21	*p* Value	RMSE
pH	5.96 ^a^	5.93 ^a^	5.98 ^a^	5.97 ^a^	6.05 ^b^	0.002	0.14
WHC%	1.38 ^a^	2.71 ^b^	2.89 ^c^	3.47 ^d^	3.98 ^e^	<0.001	0.31
L*	59.48 ^a^	61.09 ^b^	61.99 ^bc^	62.93 ^c^	61.73 ^bc^	<0.001	2.07
a*	1.82	1.06	1.31	1.49	1.42	0.122	1.04
b*	4.21 ^a^	4.68 ^ab^	5.17 ^b^	5.77 ^c^	5.52 ^bc^	<0.001	1.19
C	4.83 ^a^	4.91 ^a^	5.41 ^ab^	6.12 ^b^	5.78 ^ab^	<0.001	1.54
H	71.08	73.87	77.59	73.92	74.44	0.171	11.52

D1, 4, 7, 14, and 21 = times of storage expressed in days; WHC = water-holding capacity; L* = lightness; a* = redness index; b* = yellowness index; C = chrome; H = hue. RMSE = root square error; ^a–e^ = different letters in the same row indicate a significant difference for *p* < 0.05; data are expressed as the mean.

**Table 3:** Effect of different storage times on lipid oxidation and the sulfhydryl and carbonyl groups.

	D1	D4	D7	D14	D21	*p* Value	RMSE
TBARS (mg MDA/kg)	0.06 ^a^	0.49 ^b^	0.71 ^c^	1.27 ^d^	2.15 ^e^	<0.001	0.314
Sulfhydryl (nmol SH/mg protein)	69.11 ^e^	59.44 ^d^	52.74 ^c^	43.37 ^b^	32.27 ^a^	<0.001	5.656
Carbonyl (nmol DNPH/mg of protein)	0.67 ^a^	1.27 ^b^	1.99 ^c^	2.93 ^d^	3.62 ^e^	<0.001	0.447

D1, 4, 7, 14, and 21 = times of storage expressed in days (^a–e^ = different letters in the same row indicate a significant difference for *p* < 0.05); data are expressed as the mean.

**Table 4:** Sensory results of rabbit meat affected by different diets and PADs in different stored times.

	CNT		ELS5%		LPP3.5%				
	*n*PAD	*a*PAD	*n*PAD	*a*PAD	*n*PAD	*a*PAD	p Diets	p Pad	RMSE
Color	6.26	6.40	5.93 ^b^	6.35 ^a^	6.00	6.31	0.245	0.006	0.75
Odor	6.19	6.03	5.86	6.11	5.92	6.05	0.632	0.538	0.86
Overall acceptability	6.06	6.18	5.93 ^b^	6.22 ^a^	6.04	6.25	0.757	0.009	0.56

CNT = control diet; ELS5% = CNT diet with 5% extruded flaxseed; and LPP3.5% = CNT diet containing 3.5% extruded flaxseed and 0.2% *Padina pavonica* algae extract; *n*PAD= control pad, *a*PAD = active pad; RMSE = root square error, ^a,b^ = different letters in the same row mean a significant difference for *p* < 0.05; data are expressed as the mean.

**Table 5:** Effect of different storage times on the sensorial characteristics of rabbit meat.

	D1	D4	D7	D14	D21	*p* Value	RMSE
Color	7.31 ^e^	6.63 ^d^	6.41 ^c^	5.67 ^b^	5.04 ^a^	<0.001	0.75
Odor	7.31 ^e^	6.78 ^d^	6.24 ^c^	5.10 ^b^	4.69 ^a^	<0.001	0.86
Overall acceptability	7.18 ^e^	6.68 ^d^	6.24 ^c^	5.47 ^b^	5.01 ^a^	<0.001	0.56

D1, 4, 7, 14, and 21 = times of storage expressed in days; ^a–e^ = different letters in the same row mean a significant difference for *p* < 0.05; data are expressed as the mean.

## Data Availability

The original contributions presented in the study are included in the article/Supplementary Material; further inquiries can be directed to the corresponding author.

## Supplementary Materials

The following supporting information can be downloaded at the following website: https://www.mdpi.com/article/10.3390/foods14030404/s1, Figure S1. Normal distribution for each sensory attribute (color, odor, and overall acceptability); Figure S2. Box plot analysis for each sensory attribute to detect potential outliers of scores among panelists. Table S1. Descriptive statistics of sensory test. Table S2. Trend of physical, chemical, and sensory parameters during times for each treatment of storage.
