# Supplementary material for: Quality and Microbial Changes in Omega-3-Enriched Rabbit Meat Packaged with an Active Absorbent Pad in MAP
Source: Foods. 2025 Jan 26;14(3):404. doi: 10.3390/foods14030404 (PMC11816660; doi:10.3390/foods14030404)
Supplement: Supplementary file 1 [file foods-14-00404-s001.zip › foods-3417557-supplementary.pdf]

Supplementary Materials

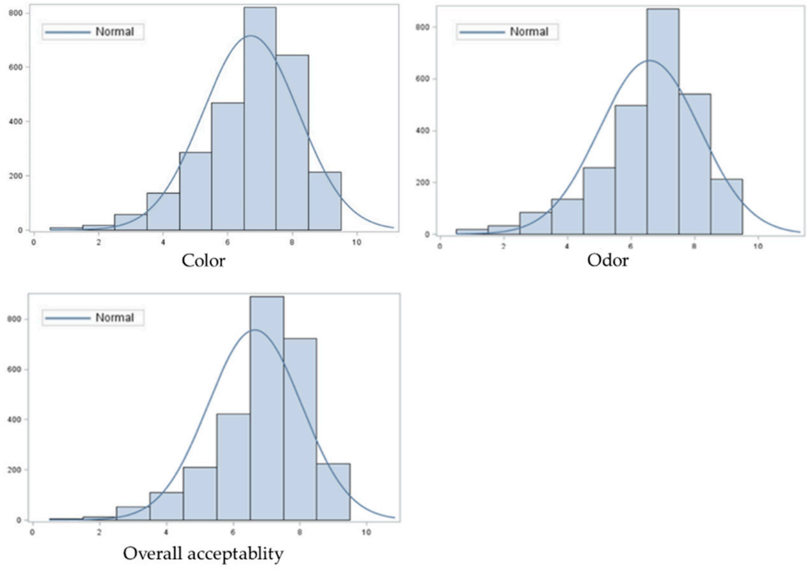

Figure S1. Normal distribution for each sensory attribute (color, odor, overall acceptability).

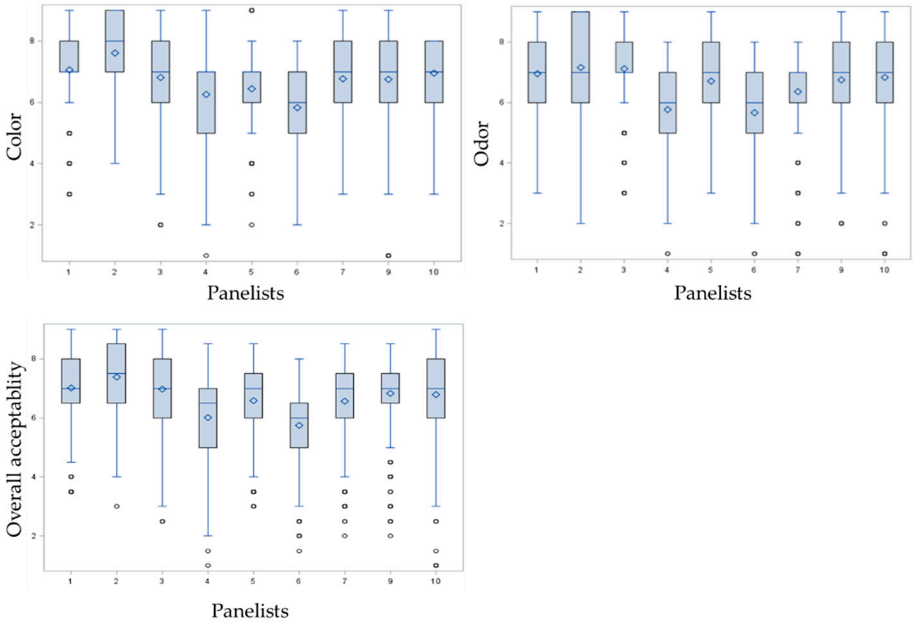

Figure S2. Box plot analysis for each sensory attribute to detect potential outliers of scores among panelists.

Table S1. Descriptive statistics of sensory test.

Variable	N	Mean	Median	Dev std	Min	Max
Color	2653	6.718	7.0	1.479	1.0	9.0
Odor	2653	6.592	7.0	1.581	1.0	9.0
Overall Acceptability	2653	6.592	7.0	1.399	1.0	9.0

Table S2. Trend of physical, chemical and sensory parameters during times for each treatment of storage.

		CNT		ELS5%		LPP3.5%	
**Parameters**	**Time day**	***n*PAD**	***a*PAD**	***n*PAD**	***a*PAD**	***n*PAD**	***a*PAD**
**pH**	D1	5.94	5.97a	5.99	6.00	5.96	5.93^{a}
	D4	5.85^{A}	6.10^{abB}	5.93^{A}	5.92^{A}	5.91^{A}	5.86^{aA}
	D7	5.94	6.07^{ab}	5.98	6.02	5.94	5.96^{ab}
	D14	5.93	6.06^{ab}	5.96	5.96	5.97	5.95^{a}
	D21	5.99^{A}	6.14^{bB}	5.99^{A}	6.02^{AB}	6.06^{AB}	6.09^{bAB}
**WHC** (%)	D1	1.47^{aAB}	1.35^{aAB}	1.33^{aAB}	1.20^{aA}	1.63^{aB}	1.34^{aAB}
	D4	2.61^{b}	2.73^{b}	2.76^{b}	2.65^{b}	2.80^{b}	2.68^{b}
	D7	3.09^{cB}	2.94^{cAB}	2.72^{bA}	2.70^{bA}	3.08^{bB}	2.82^{bAB}
	D14	3.70^{dB}	3.50^{dAB}	3.28^{cAB}	3.19^{cA}	3.72^{cB}	3.43^{cAB}
	D21	4.21^{eB}	3.86^{eA}	3.90^{dAB}	3.86^{dA}	4.15^{dB}	3.91^{dAB}
**L***	D1	59.16^{aAB}	58.02^{aA}	59.50^{aAB}	59.10^{aAB}	60.78^{aB}	60.32^{aB}
	D4	61.85^{b}	60.44^{b}	60.82^{ab}	61.35^{b}	61.81^{ab}	60.26^{a}
	D7	61.80^{b}	62.05^{bc}	61.95^{b}	61.94^{b}	62.04^{ab}	62.15^{ab}
	D14	62.30^{bA}	62.43^{cAB}	62.19^{bA}	63.20^{bcAB}	62.83^{bAB}	64.63^{bB}
	D21	59.82^{aA}	59.85^{abA}	62.43^{bAB}	63.67^{cB}	61.18^{abAB}	63.45^{bB}
**a***	D1	1.86	1.74^{ab}	2.22	1.56	1.51	1.56
	D4	1.43	1.09^{a}	1.26	1.70	0.86	0.75
	D7	1.34^{AB}	1.33^{abAB}	1.47^{AB}	1.84^{B}	0.66^{A}	1.27^{AB}
	D14	2.17^{B}	2.26^{bB}	1.57^{AB}	1.46^{AB}	0.75^{A}	1.36^{AB}
	D21	1.73^{B}	1.97^{abB}	1.35^{AB}	1.11^{AB}	0.70^{A}	1.69^{AB}
**b***	D1	3.91^{a}	3.53^{b}	4.36^{a}	4.40^{a}	4.55^{b}	4.55^{b}
	D4	4.50^{aAB}	5.09^{aB}	5.60^{bB}	5.26^{abB}	3.56^{aA}	4.07^{aAB}
	D7	4.86^{a}	4.85^{a}	5.77^{b}	5.28^{ab}	5.69^{c}	4.58^{ab}
	D14	6.32^{b}	5.67^{a}	5.52^{b}	6.17^{b}	5.86^{c}	5.47^{b}
	D21	6.50^{bC}	4.19^{abA}	5.31^{abAB}	6.11^{bBC}	5.52^{bcB}	5.51^{bB}
**C**	D1	4.51^{a}	4.25^{a}	4.85	5.26	4.98^{ab}	5.14
	D4	4.62^{aAB}	5.46^{abB}	5.90^{B}	5.52^{B}	3.81^{aA}	4.14^{A}
	D7	5.11^{a}	5.13^{ab}	6.10	5.53	5.92^{b}	4.66
	D14	6.80^{b}	6.15^{b}	5.76	6.41	6.06^{b}	5.56
	D21	6.91^{bB}	4.60^{aA}	5.53^{AB}	6.29^{B}	5.78^{bAB}	5.57^{AB}
**H**	D1	69.25	71.43	69.78	70.21	69.78	70.21
	D4	71.28	72.66	71.99	73.01	71.99	73.01
	D7	72.94	74.51	75.17	74.79	75.17	74.79
	D14	68.22	70.68	75.62	72.20	75.62	72.20
	D21	67.92	71.73	76.64	79.20	76.64	79.20
**TBARS** (mg MDA / kg)	D1	0.05^{a}	0.06^{a}	0.07^{a}	0.06^{a}	0.06^{a}	0.05^{a}
	D4	0.49^{bAB}	0.45^{bAB}	0.68^{bB}	0.54^{bAB}	0.41^{bAB}	0.34^{bA}
	D7	0.68^{cAB}	0.61^{cA}	0.93^{cB}	0.75^{cAB}	0.72^{cAB}	0.55^{cA}
	D14	1.17^{dAB}	1.02^{dA}	1.60^{dB}	1.46^{dB}	1.30^{dAB}	1.10^{dA}
	D21	2.13^{eAB}	2.07^{eAB}	2.48^{eC}	2.16^{eB}	2.16^{eB}	1.78^{eA}
**Sulfhydryl** (nmol SH / mg protein)	D1	69.16^{e}	66.91^{e}	68.81^{d}	67.31^{e}	72.13^{e}	70.35^{e}
	D4	60.07^{d}	60.29^{d}	55.62^{c}	58.47^{d}	61.70^{d}	60.46^{d}
	D7	50.46^{c}	52.92^{c}	51.84^{c}	52.22^{c}	54.40^{c}	54.57^{c}
	D14	42.51^{b}	43.35^{b}	41.69^{b}	42.47^{b}	42.94^{b}	47.26^{b}
	D21	27.19^{aA}	34.03^{aB}	33.38^{aB}	35.87^{aB}	31.49^{aAB}	31.66^{aAB}
**Carbonyl** (nmol DNPH / mg of protein)	D1	0.60^{a}	0.63^{a}	0.59^{a}	0.68^{a}	0.75^{a}	0.74^{a}
	D4	1.34^{bAB}	1.09^{bA}	1.14^{bA}	1.03^{bA}	1.44^{bAB}	1.57^{bB}
	D7	1.89^{cA}	1.81^{cA}	1.94^{cAB}	1.91^{cAB}	2.27^{cB}	2.10^{cAB}
	D14	2.83^{dAB}	2.59^{dA}	2.98^{dAB}	2.88^{dAB}	3.18^{dB}	3.11^{dB}

	D21	3.47^{eAB}	3.37^{eA}	3.83^{eB}	3.62^{eAB}	3.81^{eB}	3.61^{eAB}
**Color**	D1	7.42^{d}	7.39^{c}	7.06^{c}	7.48^{b}	7.13^{c}	7.40^{c}
	D4	6.71^{cd}	6.87^{bc}	6.45^{cb}	6.58^{a}	6.49^{c}	6.69^{bc}
	D7	6.59^{c}	6.61^{b}	5.93^{ab}	6.44^{a}	6.36^{bc}	6.50^{b}
	D14	5.74^{b}	5.81^{ab}	5.13^{a}	5.78^{a}	5.63^{b}	5.89^{ab}
	D21	4.86^{aA}	5.33^{aB}	5.07^{aAB}	5.49^{aB}	4.40^{aA}	5.07^{aAB}
**Odor**	D1	7.50^{c}	7.36^{c}	7.12^{c}	7.47^{c}	6.98^{b}	7.45^{c}
	D4	6.87^{bc}	6.76^{bc}	6.69^{bc}	6.67^{bc}	6.73^{b}	6.96^{bc}
	D7	6.36^{b}	6.21^{b}	5.95^{b}	6.37^{b}	6.30^{b}	6.25^{b}
	D14	5.20^{a}	5.19^{a}	4.94^{a}	5.19^{a}	4.97^{a}	5.13^{a}
	D21	5.00^{a}	4.62^{a}	4.60^{a}	4.87^{a}	4.62^{a}	4.44^{a}
**Overall acceptability**	D1	7.33^{d}	7.10^{c}	7.00^{c}	7.34^{c}	7.00^{d}	7.31^{c}
	D4	6.61^{c}	6.68^{bc}	6.60^{bc}	6.63^{bc}	6.65^{cd}	6.89^{c}
	D7	6.16^{c}	6.27^{b}	6.01^{b}	6.35^{b}	6.32^{c}	6.31^{b}
	D14	5.44^{b}	5.54^{a}	5.29^{a}	5.53^{a}	5.40^{b}	5.59^{a}
	D21	4.75^{aA}	5.33^{aB}	4.76^{aA}	5.25^{aB}	4.84^{aAB}	5.14^{aAB}

CNT=control diet; ELS5% = CNT diet with 5% extruded flaxseed; and LPP3.5% = CNT diet containing 3.5% extruded flaxseed and 0.2% *Padina pavonica* algae extract; *n*PAD= control pad; *a*PAD=active pad; D1,4,7,14, and 21= times of storage expressed in days; a, b, c, d, e=different letters in the same column means significant difference for $p < 0.05$; A, B, C = different letters in the same row means significant difference for $p < 0.05$; Data are expressed as mean.
